# The different perspectives of patients, informal caregivers and professionals on patient involvement in primary care teams. A qualitative study

**DOI:** 10.1111/hex.12824

**Published:** 2018-09-17

**Authors:** Kirti D. Doekhie, Mathilde M. H. Strating, Martina Buljac‐Samardzic, Hester M. van de Bovenkamp, Jaap Paauwe

**Affiliations:** ^1^ Erasmus School of Health Policy and Management (ESHPM) Erasmus University Rotterdam Rotterdam The Netherlands; ^2^ Department of Applied Economics Erasmus University Rotterdam Rotterdam The Netherlands; ^3^ Department of Human Resource Studies Tilburg University Tilburg The Netherlands

**Keywords:** patient involvement, perspectives, primary care, primary care teams, qualitative interviews, teams

## Abstract

**Background:**

Patient involvement in the decision‐making process, especially for chronically ill elderly patients, has become an important element of patient‐centred primary care in many countries, including the Netherlands. This study openly explores different perspectives of patients, informal caregivers and primary care professionals on patient involvement in primary care team interactions.

**Methods:**

Sixty‐four qualitative semi‐structured interviews with chronically ill elderly patients, informal caregivers and primary care professionals from various disciplines. Underpinned by a phenomenology approach, this study used conventional content analysis for data analysis.

**Results:**

Participants have different views of the roles of patients and informal caregivers in the primary care team and thus different expectations of the extent and level of patient involvement. Three challenges impact patient involvement in the team: (a) patients feel misunderstood and less involved that they would like when professionals take control, (b) patients have to balance the conflicting opinions of different professionals and (c) informal caregivers act undesirably as team leaders due to their own view of the level of patient involvement.

**Discussion and conclusion:**

Patient involvement is formed in complex interactions between patients, informal caregivers and multiple professionals whose perspectives and expectations can be misaligned. Recognizing the value of patients and informal caregivers on the team could help professionals to understand them better and thus limit the likelihood of challenges arising in team interactions.

## 1 INTRODUCTION

In the last two decades, health care has moved from a paternalistic professional‐centred model towards a patient‐centred care model that tailors care to patients’ needs, values and experiences.^1^, ^2^, ^3^ Patient involvement, defined as “enabling patients to take an active role in deciding about and planning their care,” is part of patient‐centred care and increasingly pursued in many countries.^4^, ^5^ The fast‐growing literature on patient involvement in the decision‐making process predominantly focuses on exploring factors that influence patient behaviour and active involvement.^6^, ^7^, ^8^

The relational aspects of patient involvement are much discussed in the literature.^6^, ^8^, ^9^, ^10^ Davis et al. show that patient involvement is influenced by the way professionals interact with patients.^8^ Moreover, Smith et al. show how relatives and friends (i.e. informal caregivers) play a key role in patient involvement, for example by collecting information on the patient's behalf.^10^

Building on such studies, this study contributes to the literature by exploring patient involvement in the decision‐making process during interactions between patients, informal caregivers and primary care professionals in primary care teams. From this perspective, patient involvement is not a clear‐cut concept, rather, it is coproduced through dialogue and interaction by patients, informal caregivers and professionals in their reciprocal relationships on the primary care team.^6^ This makes it important to focus on patient involvement *within* primary care teams.

The patient can be seen as the single binding factor of the primary care team, as actual care delivery should depend on a patient's specific wishes and needs.^11^, ^12^ Various patient involvement models see the patient as an expert with experiential knowledge of their own condition that could complement the knowledge of professionals.^3^, ^13^, ^14^ Both patients and professionals often rely heavily on informal care.^15^, ^16^, ^17^, ^18^ Informal caregivers (usually close family) are important members of the patient's support system who can provide emotional and everyday illness‐related support.^18^ However, in some cases, they can also hinder patient involvement, by being overprotective or offering more than the patient desires.^19^ Regarding patients and informal caregivers as valid members of the team alongside professionals may contribute to delivering higher quality care.^14^ However, many professionals do not regard the patient or informal caregiver as full team members and ignore their vital knowledge.^12^, ^14^ Thus patients and informal caregivers sometimes feel left out or unheard.^4^, ^20^

### 1.1 Focus and aim of the study

This study focuses on patient involvement in the decision‐making process for chronically ill elderly patients. Given the rapidly rising prevalence of these patients, their involvement is found to be particularly important.^9^, ^21^ Usually needing long‐term care, elderly patients are often supported by informal caregivers as well as primary care professionals, which lead to frequent interactions between patients, informal caregivers and multiple professionals from different disciplinary backgrounds.^11^, ^15^

The study focuses not just on one perspective (e.g. the patient) as is often the case in the literature.^4^, ^14^, ^22^ Instead, we analyse the perspectives of all three actors (i.e. patients, informal caregivers and professionals) on their interactions by not merely examining patient‐professional or patient‐informal caregiver interactions as have been studied before.^6^, ^9^, ^19^, ^23^, ^24^ We also explore the influence of interactions among multiple professionals from different disciplinary backgrounds and among multiple informal caregivers on patient involvement.

Thus, the aim of this study was to openly explore the perspectives of patients, informal caregivers and primary care professionals on patient involvement in the decision‐making process in primary care team interactions. Our research question is: *What are the perspectives of patients, informal caregivers and primary care professionals on patient involvement in the decision making process in primary care teams?* It is important to expand the knowledge on the relational elements influencing patient involvement, and the insights gained from this study could be applied to further improve patient involvement interventions in the future.

## 2 METHODS

### 2.1 Study design

We conducted qualitative interviews to collect the data. Given the aim, a phenomenology approach allowed us to gain a deeper understanding of the subjective experiences of patients, informal caregivers and professionals with patient involvement in primary care teams within their own “life‐world,” meaning the interactions between patients, informal caregivers and professionals.^25^ We followed the consolidated criteria for reporting qualitative studies (COREQ) (Table 1).^26^

**Table 1:** Report on the accordance with the COREQ checklist for reporting qualitative research

No item	Description
Domain 1. Research team and reflexivity	
1. Interviewer/facilitator	K.D. (first author) conducted all the interviews
2. Credentials	KD was a PhD student, Master of Science (Msc) in Health Care Management and Master in Law (LL.M) in Health Care Law. MS, MBS, HB and JP have a PhD
3. Occupation	KD is working as a PhD student at the Erasmus School of Health Policy and Management (ESHPM), Erasmus University Rotterdam, the Netherlands. MS, MBS and HB are working as senior researchers at the ESHPM. JP is a professor at ESHPM and at Tilburg University, the Netherlands
4. Gender	KD, MS, MBS and HB are female. JP is male
5. Experience and training	The main researcher KD had experience in quantitative and qualitative research. She received two Masters degrees from the Erasmus University Rotterdam, the Netherlands. In addition, she underwent additional formal PhD education in qualitative research
Relationship with participants	
6. Relationship established	There was no relationship between the researcher/interviewer with the patients, informal caregivers and 32 of the professionals. There was a relationship with six of the professionals. The researcher met these professionals during academic conferences or they were introduced to the primary researcher by colleagues of the research department for the purpose of this research project
7. Participant knowledge of the interviewer	The participants got the information that the interviewer was from the Erasmus University and that the research project was part of her PhD research. Also, the participants were given the information that the aim of the research was to gain more insight into their perspectives of what patient involvement is and how patient involvement is part of their daily interactions (with patients, informal caregivers and/or primary care professionals). When the participants asked, KD told more about her background as a researcher
8. Interviewer characteristics	The main interest of KD in the topic was based on previous research on the conceptualization of primary care teams and the heterogeneity of chronically elderly patients regarding their needs and wishes in their care
Domain 2: Study design	
*Theoretical framework*	
9. Methodological orientation and Theory	The underlying research paradigm for this study was phenomenology. In phenomenology, researchers are focused the “life‐world” of individuals. In this study, we explored the daily life of and interactions between patients, informal caregivers and primary care professionals. Conventional content analysis was used for data analysis
*Participant selection*	
10. Sampling	Convenience sampling and a snowball method were used. The participants were geographically spread across the Netherlands. The sampling method is explained in the article. All approached participants agreed to participate
11. Method of approach	In the convenience sampling phase, the six professionals were approached via telephone or email. The professionals were asked for contact details of other professionals suitable for this study. All professionals were asked whether they knew patients and/or informal caregivers who would be suitable for this study. The professionals were also given an information letter to give to the patients and/or informal caregivers. The contact details of the patients and/or informal caregivers were given by the professionals to the researcher by phone or email. The patients and informal caregivers were then approached by phone or email to set up an interview date
12. Sample size	In total, 64 interviews were conducted: 19 patients, 10 informal caregivers and 38 primary care professionals, The 38 professionals were 6 general practitioners, 7 physiotherapists, 15 (district) nurses, 7 occupational therapists and 3 geriatric specialized practice nurses
13. Nonparticipation	No participants withdrew from the study
*Setting*	
14. Setting of data collection	The interviews took place at a participant's preferred location. For the patients and informal caregivers, this location was their home. For the professionals the preferred location was their workplace
15. Presence of nonparticipants	At the interviews with three patients (patients 1, 2 and 13), their informal caregiver was also present. During the other interviews, no one else was present beside the participant and the researcher
16. Description of the sample	The participants’ characteristics are described in Tables 2, 3, 4
*Data collection*	
17. Interview guide	A topic list was used during the questions. Some of the questions of the topic list are given in Table 5. Because of the semi‐structured nature of the interview, the topic list was used to give guidance to the interviews but was not binding for the content of the interviews. The topic list was adjusted throughout the interviewing phase of the research
18. Repeat interviews	No repeated interviews were carried out with the participants. Regarding the patients, this was because of their age and multimorbidity. Regarding the informal caregivers and professionals, time constraints of the participant and a long distance between the participant and the researcher were the reasons for no repeated interviews
19. Audio/visual recordings	All interviews were audio recorded with consent of the participants. The recordings were stored at the first authors’ computer (KD) according to rules and regulations on data management of the Erasmus University Rotterdam
20. Field notes	KD made field notes during and after the interviews. These notes included observations and impressions that were not recorded such as nonverbal communication of the participant. Field notes were used in the analysis of the results
21. Duration	The duration of the interviews varied between 40 min and 1.5 h
22. Data saturation	Data saturation was discussed in the research team and reached for the interviews with the participants
23. Transcripts returned	Due to several practical reasons (old age of the patients and/or informal caregivers, time constraints of the participants, no possibility to use Internet connection), the transcripts were not returned to the participants for comments
Domain 3: Analysis and findings	
*Data analysis*	
24. Number of data coders	The first author performed the open coding of the data. The whole research team participated in the axial and selctive coding process. Information on the coding of the data is provided in the method section of the article
25. Description of the coding tree	No coding tree was used. The themes were derived from the data as we used conventional content analysis for data analysis
26. Derivation of themes	The themes were derived from the data and were discussed and agreed on by all the authors
27. Software	Atlas TI program was used for the coding and analysis of the data
28. Participant checking	Due to practical reasons as explained at number 23, there was no feedback of the participants on our findings. During the interviews, the researcher repeated and summarized the answer of the participant to ask for clarifications and confirmation of the interpretation of the researcher of the answers. At the end of the interview, the researcher gave a short summary of the interview content to ensure the researcher did understand the main content right
*Reporting*	
29. Quotations presented	The themes in the result section are illustrated by participant quotations. Each quotation is identified by a participant number. The participant numbers do not correspond with the numbers in Tables 2, 3, 4 to ensure the anonymity of the participants
30. Data and findings consistent	To our point of view, the presented data and findings are consistent
31. Clarity of major themes	The major themes are present in the result section of the article. Each theme is given a different heading
32. Clarity of the minor themes	Minor themes are described in the result section and addressed as subthemes of the major themes

This study defines primary care teams as a platform of interaction between patients, informal caregivers and primary care professionals. Research shows that various primary care professionals become team members depending on the course of the patient's illness and suggest that patients and informal caregivers should also be seen as team members.^11^, ^18^, ^27^ We did not examine teams as a whole (i.e. one specific patient, his/her informal caregiver and all professionals involved). Rather, we aimed to openly explore the perspectives of the potential “team members” and thus selected interviewees within one of the three participant groups. The elderly are defined as aged 60 years or older in correspondence with the World Report on Ageing and Health of the World Health Organization.^28^ We conducted in total 64 interviews with elderly patients (n = 19), informal caregivers (n = 10) and primary care professionals (n = 38) who were as follows: general practitioners (n = 6), physiotherapists (n = 7), (district) nurses (n = 15), occupational therapists (n = 7) and geriatric specialized practice nurses (n = 3). Tables 2, 3, 4 provide details of the participants.

**Table 2:** Characteristics of patients (n = 19)

Patients	Age	Gender	Chronic condition(s)	Informal caregiver	Most involved primary care professionals
1	62	Male	Paraplegic, hearing disability	Spouse	GP, (district) nurse
2	68	Female	COPD, physical limitations due to stroke	Daughter	GP, physiotherapist, (district) nurse
3	75	Female	COPD, Parkinson's disease	Spouse	GP, physiotherapist, (district) nurse
4	77	Male	Prostate cancer, limitations due to stroke	Spouse	GP, geriatric specialized practice nurse, physiotherapist, (district) nurse
5	77	Female	Stroke, rheumatic disease, heart failure	Daughter	GP, occupational therapist, (district) nurse
6	77	Female	Cardiovascular disease, rheumatic disease	Daughter	GP, physiotherapist (district) nurse
7	77	Female	Asthma, hearing disability, Parkinson's disease	Spouse	GP, physiotherapist, (district) nurse
8	78	Female	Cardiovascular disease, osteoporosis, arthritis	Friend	GP, physiotherapist, (district) nurse
9	81	Female	Asthma, hearing disability	Daughter	GP, (district) nurse
10	82	Female	Parkinson's disease, vision problems	Daughter	GP, physiotherapist, occupational therapist, (district) nurse
11	83	Female	Asthma, rheumatic disease	Son	GP, geriatric specialized nurse, (district) nurse
12	85	Female	Arthritis, limitations due to stroke	Son and daughter	GP, occupational therapist, (district) nurse
13	85	Male	Stroke, arthritis, hypertension	Daughter	GP, physiotherapist, (district) nurse
14	87	Female	Osteoporosis, heart failure	Daughter	GP, occupational therapist, physiotherapist, (district) nurse
15	89	Male	Limitations due to heart attack, vision problems	Spouse	GP; physiotherapist; (district) nurse
16	89	Female	Rheumatic disease	Daughter	GP, physiotherapist, (district) nurse
17	90	Female	Diabetes, heart failure	Granddaughter	GP, geriatric specialized practice nurse, physiotherapist, (district) nurse
18	91	Female	Multiple sclerosis, hearing disability, vision problems	Spouse	GP, occupational therapist, (district) nurse
19	98	Female	Heart failure; vision problems	Daughter	GP, (district) nurse

GP, general practitioner.

**Table 3:** Characteristics of informal caregivers (n = 10)

Informal caregivers	Age	Gender	Relationship to patient
1	57	Female	Daughter
2	60	Male	Daughter
3	65	Female	Spouse
4	71	Female	Spouse
5	73	Female	Spouse
6	75	Male	Spouse
7	77	Male	Spouse
8	77	Male	Spouse
9	79	Male	Spouse
10	87	Male	Spouse

**Table 4:** Characteristics of primary care professionals (n = 38)

	Age	Gender	Number of years as professional employment
General practitioners			
1	34	Female	3
2	40	Female	15
3	43	Male	10
4	44	Female	16
5	57	Female	35
6	58	Male	32
Physiotherapists			
1	24	Female	1.5
2	31	Female	9
3	34	Male	34
4	37	Female	20
5	41	Male	14
6	51	Female	30
7	63	Female	39
(District) nurses			
1	23	Female	2
2	27	Female	2
3	29	Female	4
4	32	Female	16
5	33	Female	10
6	34	Female	12
7	42	Female	15
8	46	Female	12
9	46	Female	17
10	54	Female	16
11	55	Female	33
12	55	Female	30
13	55	Female	16
14	55	Female	30
15	57	Male	35
Occupational therapists			
1	25	Female	1
2	28	Female	4
3	32	Female	10
4	34	Female	16
5	35	Female	16
6	36	Female	17
7	62	Female	41
Geriatric specialized practice nurses			
3	40	Female	8
1	41	Female	10
2	59	Female	13

Our study protocol (No. MEC‐2017‐207) was reviewed by the medical ethics committee of the Erasmus Centre, Rotterdam, the Netherlands. The Medical Research Involving Subjects Act did not apply, so the committee waived further examination.

### 2.2 Data collection

The first author (i.e. KD; primary researcher) collected the data. Prior to the study, the researcher had no established relationships with the participating patients, informal caregivers and 32 of the 38 primary professionals. First, convenience sampling was used to select six professionals. Selection criteria were (a) working as one of the five types of primary care professionals and (b) involved in caring for chronically ill elderly. The primary researcher first approached six professionals in her own network (i.e. from previous research projects or introduced by a colleague researcher) via email or telephone. Then, a snowball method was used. That is, during the interviews with these six, the researcher asked for the contact details of other professionals who would be suitable to take part in this study. These 32 professionals were invited to be interviewed via telephone and email and all agreed. At the interviews, the professionals were given a letter about the purpose of the study to pass on to patients and informal caregivers who would also be suitable for this study, asking for their consent to be contacted by the researchers. Subsequently, the people who consented were approached by telephone or email and all agreed to take part. Interviews lasted until no new insights were offered (i.e. data saturation).

### 2.3 Interviews and study procedure

The interviews took place at the participant's preferred location and lasted between 40 and 90 minutes. The informal caregiver of patients 1, 2 and 13 was also present during the interview. The interview began with the researcher introducing herself to the participant, explaining the reasons for doing the research and asking for explicit verbal consent to audio record their conversation. Informed consent was assumed by participants’ agreement and completion of the interview. All participants gave permission to use quotations from the interviews anonymously. At any time, respondents were allowed to withdraw their consent and end the interview. None withdrew their consent.

The semi‐structured interviews were conducted in person. The primary researcher developed the topic lists and interview guides and revised these following inputs from the entire research team. The interviews focused on the interactions in primary care teams and covered three main topics: (a) participants’ perspectives on primary care teams and team membership (b) differences in the nature and level of involvement in the team and (c) the role of professionals and informal caregivers in stimulating or hindering patient involvement in the team. All the participants were invited to illustrate their answers from real‐life situations. Table 5 provides a selection of questions asked in the interviews.

**Table 5:** Main interview topics and questions

	Questions		
Topics	Patients	Informal caregivers	Primary care professionals
1. Participants’ perspectives on primary care teams and team membership	(a) Please describe the people involved in your care process	(a) What does the word “primary care team” mean to you?	(a) What does a primary care team mean to you?
	(b) What activities do you do to benefit your health?	(b) Who do you consider to be part of the primary care team of your family member?	(b) Please list who you consider a member of your primary care team?
2. Differences in the nature and level of involvement between patients	(a) Please describe how decisions concerning your health are usually made.	(a) How well can your family member make decisions about their own treatment?	(a) Have you come across any differences in the level of patient involvement and if so, what kind?
	(b) Have you ever disagreed with a family member or professional on your care team? If so, what did you do?	(b) How well can your family member fully understand their health situation?	(b) Please give examples of (1) a patient highly involved in their care process and (2) a patient not involved in the process.
3. The role of professionals and informal caregivers in stimulating or hindering patient involvement	(a) What do you find important in the care you receive from this person [professional or informal caregiver]?	(a) How would you describe your own role in looking after your family member?	(a) How would you describe your professional role in stimulating patient involvement?
	(b) Is there anything you wish was different in the way you receive care from this/these person/s?	(b) How would you describe the interaction or relationship with [a professional]?	

This table provides insight into some of the questions posed in the interview guide.

### 2.4 Data analysis

The interviews were transcribed verbatim and analysed with Atlas TI. Given the explorative nature of this study, conventional content analysis was used, with the themes derived from the data and not based on preconceived categories or theoretical perspectives.^29^ KD first openly coded the data, whereupon MS, MBS, HB and JP and KD (i.e. the whole research team) performed axial coding, grouping comparable codes into one code. For example, the codes “hesitant to speak up to a professional” and “difficulties sharing feelings with a professional” were grouped together under “patient's ability to speak to professionals.” Then, the research team discussed the codebook and performed selective coding, which led to two major themes: (a) who is considered part of the team and (b) challenges in the team that (could) impact patient involvement.

### 2.5 Trustworthiness

Several steps were undertaken to comply with the five quality criteria for trustworthiness of qualitative research (i.e. credibility, transferability, dependability, confirmability and reflexivity).^30^ To enhance the credibility of the results, participants were explicitly encouraged to back their views with concrete examples. Follow‐up questions were asked to explore the context of examples and enrichen the data (i.e. prolonged engagement).^30^ We used investigator triangulation, meaning that all the authors of the study discussed the axial and selective coding process as well as the analysis and interpretation of the data.^30^ Regarding the transferability of the results, the thick description used where appropriate in the Results section provides more insight into the specific context.^30^ For example, some results specifically apply to elderly patients with deteriorating cognitive abilities; this is made clear. Regarding the dependability and confirmability of the results, KD made an audit trail, which described in detail all the steps undertaken from the start of the project to the reporting of the findings.^30^ Last, to enhance reflexivity, KD kept a diary on the conceptual lens, the assumptions and preconceptions of the researchers and how these could affect the phases of the research project.^30^ The whole research team frequently discussed this diary during data analysis meetings.

## 3 RESULTS

Here, we first report on the participants’ ideas on team membership and what their role in the team is or should be. Next, we explore the various perspectives and expectations of the latter that can cause challenges within the team.

### 3.1 Who is considered part of the team?

Overall, the position and role of professionals were not contested, whereas the respondents did have diverging perspectives on the role and position of patients and informal caregivers. No patients or informal caregivers specifically mentioned either themselves or the other as part of the team. Corresponding with the professionals’ view, teams were described in terms of a professional collaboration. Especially, physiotherapists and occupational therapists saw involving patients as an essential element of their work. Patient involvement was described as “placing patients and their wishes central in the care process,” or “letting patients make their own decisions.” The views of professionals differ on whether such involvement implies that patients actually play a role *on* the team: some professionals feel that patients are team members while others acknowledge the importance of focusing on a patient's desires but still place the patient outside the team.

I don't believe that patients have a very big role. Well, it is big, in the sense that a patient's questions, care needs, wishes and limitations are the starting point, but after all that is clear, you only consult with your [primary care] team. And afterwards, you report the outcome back to the patient. (Occupational therapist 1)

Some professionals do consider informal caregivers a part of the team. Geriatric specialized practice nurses and occupational therapists see informal caregivers as key persons in the care process, often providing emotional support to patients, encouraging self‐management and taking over care tasks. Although none of the informal caregivers specifically identified themselves as team members, the majority expressed feeling highly involved in the care process and emphasised their close connection with the professionals who frequently ask them for help. This applies particularly to participants who have been informal caregivers for a number of years.

I think they [the nurses] feel that I fit in with them. I've been an informal caregiver for so long and I do so many things. I think they see me as one of them. So our relationship is very good. They also tell me things about their personal situation. It's a bit like family. (Informal caregiver 1)

A majority of the patients expect the general practitioner to lead the team. The older general practitioners particularly (i.e. 50 years or older) share this view and feel that they need to take on a steering role.

I think the older generation does not feel the need to have a clear leading role in the sense of ‘I want to be involved in the entire process’. It's more like, ‘If you say so, doctor, we will do that’. And of course you will discuss the important things. But overall, we are pretty steering. (General Practitioner 1)

Although professionals do not explicitly mention the patient as part of the team, most believe that in an ideal world patients should take a leading role in their own care process. Patients should take responsibility for their own health and only consult professionals when necessary. When patients are unable or unwilling to fulfil this role, many professionals view the informal caregiver as a proxy for the patient and expect them to step in and take the lead. Most informal caregivers try hard to involve their family member, even if he or she is less capable of fully understanding their situation. For example, some informal caregivers always have their family member join a meeting with professionals, even if their family member is not able to engage actively and the informal caregiver needs to take the lead.

### 3.2 Challenges in the team that could impact patient involvement

Our findings reveal that when ideas on the team positions and role divisions do not align, challenges can arise. These challenges impact patient involvement and the role patients can or are willing to play in their care. In the following sections, we discuss these challenges.

#### 3.2.1 Patients as active participants or passive bystanders

First, when professionals consider themselves the central figure in the team, this can negatively impact their relationship with those patients who want to play a more active role. Some patients feel limited in taking on an active role because of their interactions with professionals. They feel treated like passive bystanders in their own care process and that the professionals make decisions *for* them instead of *with* them. These patients want to be actively involved and feel obliged to express this explicitly.

The experts talk about you as if you're not even there. I always think that you should be assertive. You should tell them, like ‘hey, listen up, you know, you're talking about me.(Patient 1)

Other patients want to express their own opinions and wishes but hesitate to do so because of possible negative reactions. Patients sometimes feel that professionals do not always value their opinion, while in some situations, they feel they know best.

Most occupational therapists and physiotherapists say that is it important to encourage patients to express their wishes and make sure that the patients’ wishes are the starting point of the caregiving process. However, some of these professionals tend to fall into a “repair‐reflex” mode, immediately coming up with what they think is the best solution for a patient's problem without asking the patient what he or she believes would be best.

I think we [professionals] should say ‘Oh, I can fix that for you’ less often. I tend to do it and sometimes realize that I am patronizing them [patients]. I shouldn't. Caregivers should be more aware of this. I think that professionals today are very comfortable fixing things for people.(Geriatric specialized practice nurse 1)

#### 3.2.2 Conflicting ideas amongst professionals in the team

Second, the various professionals on the team can have conflicting ideas about the desirable level of patient involvement and their role in stimulating it. They can have diverging expectations of how professionals from other disciplinary backgrounds should act in the best interests of the patient. For example, some physiotherapists feel that helping assistants from home care organizations tend to “over help” patients, whilst physiotherapists strive to activate patients to a maximum.

A home care nurse puts the food in the microwave, brings it to the table and sets it in front of the patient. These people mean well and give lots of tender loving care. But I tell them [home care assistants]: ‘Let them [patients] get their own food out of the kitchen or at least let them bring their plate back to the kitchen’. But they [home care assistants] feel like, ‘But it only takes a second fosr us to do it’(Physiotherapist 3)

For patients, balancing the sometimes conflicting opinions of different professionals can be difficult. Besides challenges that occur daily, as illustrated in the quote above, having to deal with multiple conflicting messages can make patients lose sight of their treatment plan. Most professionals feel this applies especially to patients with low or deteriorating cognitive abilities. As the next quote illustrates, this may also lead to negative effects for professionals.

Patients often say, ‘The GP said so‐and‐so’. And then I find out it's not true and I'm like, huh? So there's lots of confusion because everyone has their own idea, […] the caregivers and the client as well. And if it isn't coordinated properly you get situations where clients say, ‘The GP told me I'll be getting physiotherapy twice a week’. Then I say, ‘Well, it's not up to the GP to decide this, it happens in consultation with me’. So you notice that we [professionals] are being played off against each other, just because things aren't clear. (Physiotherapist 2)

Different ideas about who is the central figure in the team can also cause challenges between professionals. This often has to do with patients’ central focus on the GP, which can again impact the active role patients actually or are willing to play in their care. The “Doctor knows best” attitude can cause challenges between patients and other professionals when the patient values the professional's opinion less than the GP's. Then, professionals other than GPs face the challenge of convincing the patient of the necessity of a specific treatment, as the next quote illustrates.

I see that elderly patients are very focused on authority. If I say ‘you're allowed to move around’ and the patient tells me ‘No, the doctor told me not to move’. I can jump high or low, it won't make any difference. The doctor has a higher position in the hierarchy.(Occupational therapist 2)

#### 3.2.3 Informal caregivers as undesirable leaders of the team

Third, challenges can arise when informal caregivers attribute a central role to themselves while patients have different ideas on this. Some informal caregivers act independently without involving the patient. This could be because the patient is no longer capable of understanding their situation, leaving the informal caregiver in charge. However, some informal caregivers tend to act on what they believe the patient wants without verifying their thoughts with the patient. In these situations, informal caregivers could take the lead in conversations with professionals, while the patient would have liked to make his own decisions.

For people who get lots of informal caregiving, I see their informal caregiver wants to set the care goals. Daughters, especially, bypass their parents. They just say, ‘I'd like my mother to walk again’, but they don't realize that their mother might not ever be able to walk again. Meanwhile, mother is sitting there, looking at me, like ‘walking is not my first priority’. (Physiotherapist 3)

Also, informal caregivers can be overprotective of family members, which cause them to go against professional advice. Some children believe that their parents have a right to more intensive care either the professionals or the patients feel is desirable or required. This creates challenges for patients to express their own wishes and also challenges for professionals to deal with this kind of behaviour in informal caregivers.

Some informal caregivers feel that their parent doesn't get enough care and is entitled to more. So they defend their parent's right to care. They ask you ‘What is that ointment for?’ When you explain, they say, ‘But I read this and that on the Internet, so you're wrong’. So then you tell them, ‘No, it's not wrong, it has the same effect’. They don't have a professional background, and that can cause lots of confusion between us.(District nurse 1)

Challenges can be even greater when patients receive support not from one informal caregiver, but a group of them. Often in the parent‐child caregiving relationship, elderly patients receive care and support from all their children whose opinions may not always align.

## 4 DISCUSSION

In this study, we openly explored the perspectives of patients, informal caregivers and primary care professionals on patient involvement in the decisionmaking process in interactions in primary care teams. Adding to the literature showing that patient involvement depends on the quality of the relationships between patients, informal caregivers and professionals,^8^, ^9^, ^10^ our multiperspective study reveals that misalignments in both views and expectations of the role division influence interactions and patient involvement accordingly. Patient involvement is a relational process, shaped in a context of reciprocal relationships between patients, informal caregivers and professionals.^6^ Professionals do not often consider patients and informal caregivers to be part of the team.^12^, ^14^ However, viewing patients and informal caregivers as team members is important for delivering high‐quality care, as some patients and informal caregivers have vital experiential knowledge and can therefore play crucial roles in the care‐provision process.^3^, ^13^, ^14^ Recognizing the roles of both patients and their informal caregivers in the team could help professionals to understand and collaborate better with them and thus limit the likelihood of challenges occurring in their interactions.

### 4.1 Challenges within the team

This study found three challenges caused by different perspectives and expectations of patient involvement in the primary care team. The first challenge is that professionals tend to consider themselves as the team leader and fall into a “repair‐reflex,” which may lead patients to feel misunderstood and less involved in the team than they would like. Research on self‐management of patients finds a similar repair‐reflex in home care nurses.^31^

The second challenge is that patients need to balance the sometimes conflicting opinions of multiple professionals. Research of Doekhie et al shows that primary care professionals have misaligned views on who is the most important person in the care for a patient.^11^ General practitioners often consider themselves as the key figure and physiotherapists and occupational therapists, for example, as less important, while the latter two professionals do find themselves important figures in the care process.^11^ Following this research, our study shows that the professional's idea of who the key figure in the team is and whose opinion should be leading could lead to challenges that impact patient involvement.

The third challenge concerns the role of informal caregivers in the team, and how they may have a different opinion than patients and professionals of the (desired) level of patient involvement in the team. This may prompt informal caregivers to take over the lead in the team.^19^ The expectations of patients and professionals on a patient's responsibilities and abilities may be in alignment, but their actions would be hindered by a dominant informal caregiver who has opposing or deviating expectations of what the responsibility of their loved one should be.

Although aligning the expectations of patients, informal caregivers and professionals could be seen as a scenario worth pursuing, doing so could also mean that a patient would prefer to be less involved than others may think. This notion challenges the underlying assumptions of current health policies in various countries. In Thompson's taxonomy of patient involvement, the desired levels of patient involvement range from autonomous decision making to noninvolvement and the actual level is influenced by the relationship between patients and their caregivers as well as the patient's own capacity (e.g. cognitive ability).^6^ From a policy perspective, patient involvement is highly valued and should be pursued.^4^, ^5^ Patients are encouraged to make autonomous decisions and noninvolvement is considered undesirable. Paradoxically, however, this decision may also include patients’ noninvolvement in their care process, or put differently, a strong desire to place decision making in the control of their informal caregivers and primary care professionals.^31^ The question then becomes whether active patient involvement should be imposed on those patients who want to remain passive. From our perspective, patient‐centred care implies accepting that patients have distinct preferences in the level and type of involvement, which may change over time and also depend on their current ability.^9^ Actual involvement of patients in the decisionmaking process is shaped on the microlevel in teams of patients, informal caregivers and professionals.

### 4.2 Limitations

Our study on patient involvement looked solely at chronically ill elderly patients and this should be considered when interpreting the results. However, other research shows that the level of patient involvement also differs in younger and not chronically ill patients and is also influenced by the quality of the relationships with care providers.^6^ This suggests that our findings are still generalizable to other patient groups.

Patients and informal caregivers were selected on the basis of recommendations of the professionals and not at random. Because of this, we could have potentially excluded patients and informal caregivers who are less willing or able to speak openly, but who might have had interesting insights into the interactions of the team. However, our patient group differed in their extent of preferred and actual involvement and our informal caregiver group differed in their extent of stimulating or hindering patient involvement. As a result, we were able to examine several types of interactions and relationships between actors, which provided us with a broad insight into the sometimes conflicting perspectives and expectations of all the actors concerned with patient involvement in the team decisionmaking process.

The relatively low number of interviews per respondent group could be seen as a limitation. However, data saturation was reached. Also, the purpose of our study was to openly explore patient involvement in the primary care team, and so we tried to include as many different perspectives as possible to gain broad insight. For the same reason, we did not select primary care teams as a whole (i.e. one specific patient, his/her informal caregiver and all professionals involved in the care for that patient). Therefore, we cannot draw conclusions on patient involvement in specific teams of patients, informal caregivers and professionals. Future research could focus on exploring patient involved in specific teams.

### 4.3 Implications for practice

Our study shows that (mis)alignments in expectations of the roles and responsibilities of patients, informal caregivers and professionals influence patient involvement in the team. For patient involvement, it is important that professionals and informal caregivers acknowledge that the patient is indeed a part of the team. To achieve this recognition, a first step could be to clarify what the primary care team does and who its members are. Research shows that primary care professionals, viewing the roles of their professional colleagues, regard primary care teams as fluid entities with an inner and outer layer.^11^

Our study indicates that patients may receive informal and professional care from various individuals. Therefore, the patient could be the single binding factor of the team and thus their primary care team should be conceptualized from the patient's perspective.^12^ To conceptualize primary care teams from a patient's perspective, the “concentric circles of importance” could be used for the chronically ill elderly.^18^ In this method, participants are asked to identify and describe the individuals involved in their care process and to value the importance of their role in various health‐related activities.^18^ This method determines the different layers of the primary care team.

Moreover, previous research on teams has identified role clarification (i.e. understanding the mutual roles and responsibilities of team members) as an important factor that influences the effectiveness of a team.^32^, ^33^, ^34^, ^35^ To achieve role clarification, it is important to develop positive interpersonal relationships, based on the opportunity to build trust and respect.^35^ In line with other research, we therefore suggest that patients could benefit from a meeting with their informal caregivers and involved professionals especially to discuss their preferences and abilities.^14^, ^20^ The presence of patients and informal caregivers at team meetings is shown to be appreciated by patients and professionals.^14^ Role clarification is especially important for patients with multiple chronic conditions as a wide range of different primary care professionals could be involved in their care process, each having a different perspective on patient involvement.^11^, ^14^

To compensate for hindering factors such as time constraints and geographical distance, role clarification regarding patient involvement could be integrated into existing regular interprofessional care‐planning meetings. The use of modern virtual communication technologies, such as video‐calling, would especially benefit geographically dispersed patients, informal caregivers and professionals so that these individuals could follow meetings without needing to be physically present.^36^, ^37^

## 5 CONCLUSION

Patient involvement could be enhanced by considering the individual perspectives and expectations of patients, informal caregivers and primary care professionals. In the primary care setting, patient involvement is not up to the individual patient or the result of bi‐directional relations between one patient and one informal caregiver or professional. Rather, it is shaped in the complex interactions between patients, informal caregiving and various primary care professionals whose perspectives of patient involvement may diverge greatly.

## CONFLICT OF INTEREST

All authors declare to have no conflict of interest.

## AUTHORS CONTRIBUTIONS

KD, MBS, MS and JP designed the study. KD collected the data and initiated the analysis of the data. All authors frequently discussed the appropriate methods for analysis and results of the analysis. KD in interaction with MBS, MS, HB and JP came to a final set of codes and themes. KD drafted the first version of the manuscript and based on input and reformulations of sentences of MBS, MS, HB and JP, KD revised the manuscript. All authors read and approved the definitive version of the manuscript and agree to be accountable for all aspects of the study.
